# Crude extracts of *Sesamum Indicum* roots used as anthraquinone source effect on pulping with sodium hydroxide of Sudanese bagasse

**DOI:** 10.1186/s13104-019-4075-9

**Published:** 2019-01-15

**Authors:** Suhair Kamalaldeen Shomeina, Osman Taha Elzaki, Sakina Yagi, Salaheldin Dafaalla Mohieldin, Tarig Osman Khider

**Affiliations:** 1 Cellulose Chemistry and Technology Research Unit, National Centre of Research, Khartoum, Sudan; 2 Faculty of Science, Botany Department, University of Khartoum, Khartoum, Sudan; 3 College of Applied and Industrial Sciences, University of Bahri Sudan, P.O. Box 1606, Khartoum, Sudan

**Keywords:** *Sesamum Indicum*, *Anthraquinone* (AQ), Anthrasesamones, Cooking with sodium hydroxide, Bagasse, Pulp yield, Kappa number

## Abstract

**The objectives:**

The work was carried out for extraction of natural anthrasesamones from roots of *Sesamum Indicum* using different organic solvents and utilization of extracts as catalyst in pulping with sodium hydroxide for a by-product of sugar industry (Sudanese bagasse).

**Results:**

*Sesamum Indicum* roots when extracted with ethanol, it gave the highest extracts yield % (0.964), followed by ethyl acetate, chloroform, dichloromethane and petroleum ether extracts. The chemical pulping of Bagasse was done by using of sodium hydroxide, sodium hydroxide with anthraquinone, and sodium hydroxide with extract instead of anthraquinone keeping constant conditions at temperature 160 °C and applied sodium oxide 10.9% and time was 120 min, gave promising screened yield between 49.84 and 53.68%, bleachable kappa number between 15.57 and 8.26 for sodium hydroxide only and cooking with sodium hydroxide with anthraquinone. Cooking with sodium hydroxide of bagasse with anthrasesamones gave good pulping yields and kappa number.

**Electronic supplementary material:**

The online version of this article (10.1186/s13104-019-4075-9) contains supplementary material, which is available to authorized users.

## Introduction

**Fig. 1:**
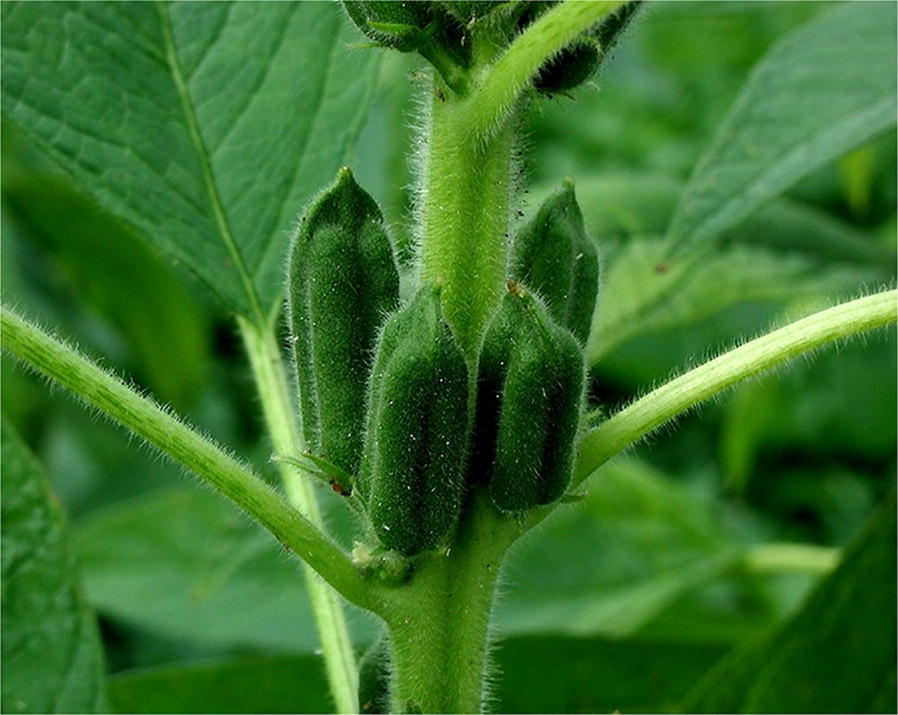
General morphology of *Sesamum Indicum* http://commons.wikimedia.org/wiki/Main_Page

Sesame (*Sesamum Indicum* L.), a member of the family Pedaliaceae, is widely grown in Sudan under rain fed conditions. The average seed yield in Sudan is about 350 kg/ha. It is one of the most important cash crops. Sudan (38.2%), India, Myanmar and China are the most important sesame producers with 68% of the world production [1] According to Encyclopedia Britannica “sesame, called Benne, it is an erect, annual plant (Fig. 1) [2].

Sesame (*Sesamum Indicum* L.) is known as sim–sim (Sudanese), till (Hindi), hu ma (Chinese), sesame (French), goma (Japanese), gergelim (Portuguese) and ajonjolí (Spanish). It is also considered to be a beneficial for food and health [2, 3]. 100 g of sesame provides 100% of the recommended daily allowance (RDA) for manganese and potassium, 57–65% of the RDA of phosphorus and iron, and 13–35% for zinc, calcium (helps to prevent colon cancer and osteoporosis) and copper **(**reducing pain and swelling of rheumatoid arthritis) are recommended daily intake is 25 to 50 g [4, 5].

Sesame seeds are used in baking and making candy while its oil, in addition to cooking, is used in the manufacture of soaps, paints, perfumes, cosmetics, and hair oils. Sesame oil has many pharmaceutical activities like antioxidant, antibacterial, cardio tonic, and anti-diabetic [6], hypocholesterolemic [7] antitumor [8, 9], antiulcer [10], and anti-inflammatory [11]. These uses are due to its antioxidant properties which accounted for by the presence of secondary metabolites. Secondary metabolites purified from seeds and roots of sesame include sesamol, sesaminol, sesamin, sesamolin, naphthoquinone hydroxysesamone and 2,3-epoxysesamone and anthrasesamones A–F. Some of these chemicals are reported to have antimicrobial properties [4, 12, 13]. In Nigeria, the leaves and roots of sesame plant are used for treating migraine, hypertension, ulcer, constipation, chicken pox and pile [12, 14]. The roots of *S. indicum* produced five unusual anthraquinones (Anthrasesamones A, B, C, D and E) having C6 side chains at C-2 in the anthraquinone ring [15]. In particular, anthrasesamone C (6) is a chlorine-containing anthraquinone, which is a rare metabolite in higher plants [3, 4].

Anthraquinones (AQ) are natural pigments that are found in some plants, fungi, lichens and some insects [16, 17]. Anthraquinones are widely applied in medicine, food and dye industry [18]. Anthraquinone is an important catalyst that is used as an additive in chemical alkaline wood pulping for its effectiveness in accelerating delignification, lowering carbohydrate degradation and improve pulp yield [16, 19, 20]. Anthraquinone will reduce the active alkali consumption, increase the degree of delignification, and increase the yield, the addition of about 0.1% anthraquinone on wood base results in a 1–3% increase in pulp yield [19].

The mechanism of anthraquinone functions in a redox reaction. anthraquinone cycles between its insoluble oxidized form and its soluble reduced form. First, anthraquinone reacts with the reducing group of a carbohydrate to be more stable against alkaline peeling reactions and producing the reduced soluble form of anthrahydroquinone. Then, the anthrahydroquinone (AHQ) reacts with the quinone methide segment of the lignin polymer increasing the rate of delignification. The anthrahydroquinone (AHQ) is also converted back to anthraquinone (AQ) at the same time and finishes a cycle reaction [21]. Utilization of anthraquinone (AQ) in sodium hydroxide cooking of bagasse, it accelerates the delignification to produce higher yields, lower Kappa numbers better mechanical properties of pulps similar to Kraft pulp grades [22, 23].Sudan is rich country with pulp and papermaking raw materials that include non-woody plants, agricultural residue, recycled papers, as well as hard wood species [24].

The present work aiming to extract of natural anthrasesamones from roots of *Sesamum Indicum* applied with different organic solvents and utilize it as anthraquinone (AQ) catalyst in cooking with sodium hydroxide of Sudanese bagasse for production of pulp paper making.

## Main text

### Methods

About 20 kg of bagasse were transported from Sugarcane Factory (Additional file 1: Figures S1, S4, S5) (White Nile State—Sudan) to National Centre of research, Cellulose Chemistry and Technology Research Unit in 2014 by bus according to Technical Association of Pulp and Paper Industry standards TAPPI [25]. Bagasse was prepared accordingly by drying in air followed by screening to remove the dirt and dust by dry depithing and screened by standard sieve. Matured *Sesamum indicum* (Simsim) roots were collected from White Nile state Roots were ground in a standard star mill with a standard sieve and closed till used. The plant species were authenticated by Dr. Hayder Abd-Algadeer Assistant Professor in Herbarium of Medicinal and Aromatic plants Institute, National Centre for Research.

Ethanol, ethyl acetate, chloroform, petroleum ether and dichloromethane were selected to extract the chemical components from *Sesamum indicum* roots (Additional file 1: Figure S2) according previous studies [18, 26–29]. The other chemicals used were potassium permanganate, potassium iodide, sodium thiosulphate, sodium hydroxide pellets purified, potassium hydroxide and starch soluble from Chemical Limited Poole England. Sulphuric acid 98% and ammonia solution from Romil Pure Chemistry. Anthraquinone from Prolabo. The chemicals were used in pulping process, determination of kappa number and identification of anthraquinones.

The presence of the anthraquinones in the *Sesamum indicum* roots was tested by using two grams of milled roots followed by boiling with 0.5 N potassium hydroxide KOH, and addition of 10 ml of chloroform; then shaking and the filtration was done. Addition of 3 ml ammonia with 10% concentration was done, the red pinkish color in ammonia was sign presence of anthraquinones according to Neeta [4].

The extraction with ethanol, ethyl acetate, chloroform, petroleum ether 60–80 and dichloromethane was done separately for 30 gm of *Sesamum indicum* roots using a Soxhlet extraction unit, the crude extracts were evaporated to dryness on rotator evaporator.

Cooking with sodium hydroxide, sodium hydroxide with anthraquinone (AQ) and sodium hydroxide with crude extract containing anthraquinone were applied in 7-litre rotary electrically heated digester. Bagasse was cooked according to following conditions, Active alkali as sodium oxide Na_2_O was 10.9%, Anthraquinone (AQ) concentration 0.1%, liquor to bagasse ratio 5:1, with maximum temperature 160 °C, time to maximum temperature 120 min and time at maximum temperature 90 min. Pulping liquors were added directly at the start of cooking. Determination of moisture content of each pulp was done, the total yield (combination of screened yield and rejects) were calculated. Kappa number (degree of delignification) was carried out according to Technical Association of Pulp and Paper Industry standards (TAPPI) T236 om-99.

### Results

All extractives under investigation gave positive results thus extract with ethanol, ethyl acetate, chloroform, dichloromethane and petroleum ether 60–80 gave 0.964%, 0.876%, 0.664%, 0.605% and 0.264%, respectively and this agreed with Furumoto and his research work group [15].

Bagasse was chosen due to the fact that it was Industrial agricultural waste and mostly utilized for energy supply in sugar and ethanol mills as well as easy to be cooked for bleachable kappa number. It was cooked with sodium hydroxide and sodium hydroxide with AQ cooking with yields presented in Table 1 depending on Karar [30]. Application of sodium hydroxide only at maximum temperature 160 °C for 2 h and active alkali from 10.9% as Na_2_O gave bleachable Kappa number and screened yield 49.8%. Anthraquinone (AQ) is a powerful redox-catalyst in alkaline pulping especially when non-woody raw material is cooked [31, 32], with addition of 0.1 AQ at same cooking conditions higher degree of delignification (kappa number 8.26) was attained associated with increase in screened yield (53.7%). It well known that the addition of AQ accelerated the delignification and preserved the carbohydrates [33–37].

**Table 1:** Pulping properties of sodium hydroxide and sodium hydroxide with anthraquinone of bagasse

Conditions	Soda	Soda AQ
Active alkali as Na_2_O%	10.9	10.9
Liquor to bagasse ratio	5	5
Maximum temperature, °C	160	160
Time at maximum temperature, min	120	120
Screened yield%	49.8	53.7
Reject%	0.3	0.4
Total yield%	50.1	54.1
Kappa No.	15.8	8.3

The results achieved by using *Sesamum Indicum* roots crude extractives as AQ source applying to cook bagasse with soda according to the reference cooks, showed that, the most promising findings were achieved by pulping with dichloromethane extracts (Additional file 1: Figure S3), which obtained highest screened yield (50.5%), satisfactory kappa number (19.4) and negligible rejects (1.64%) and this agreed with Furumoto [15].

**Fig. 2:**
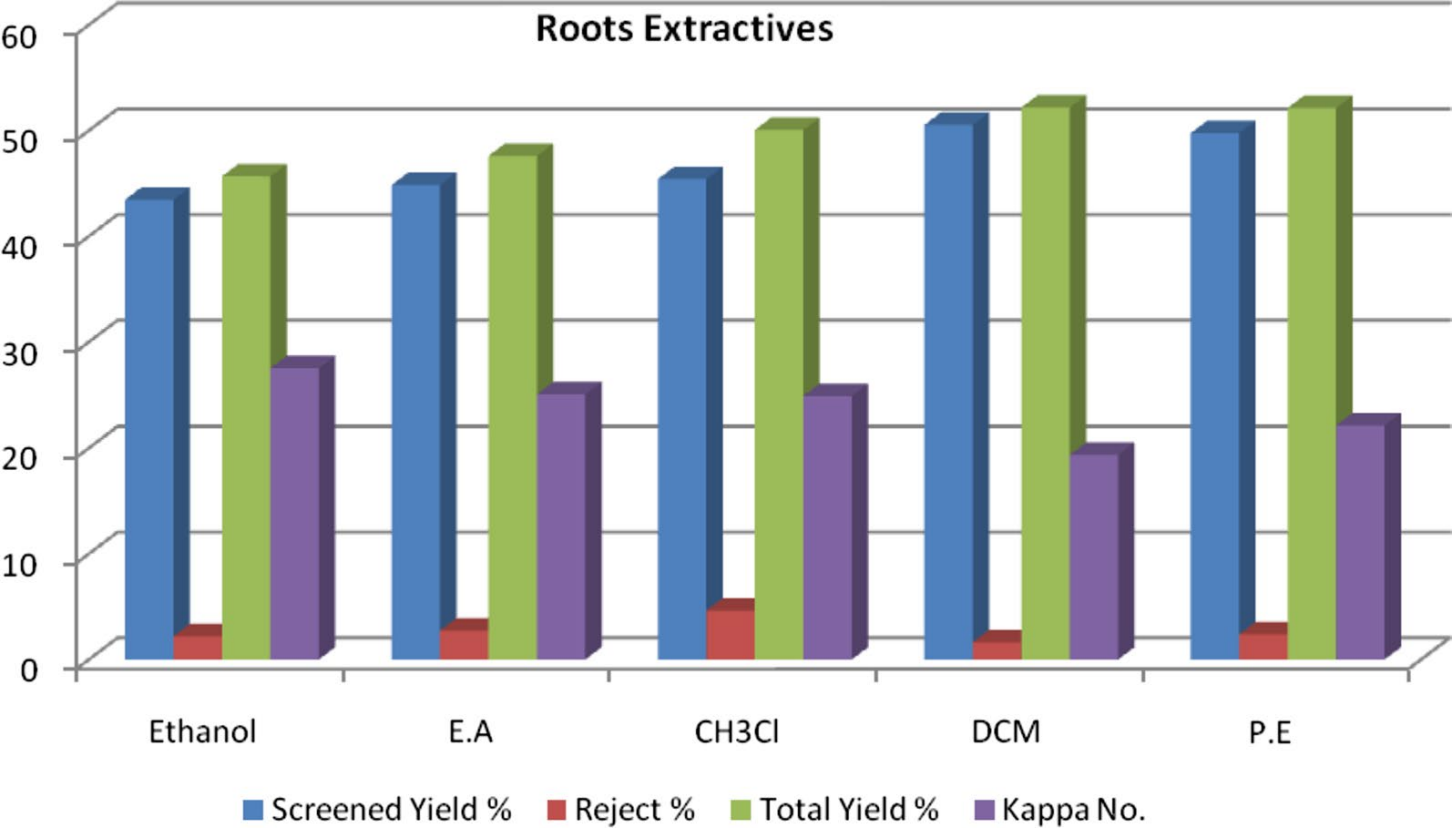
Pulping results for *Sesamum Indicum* roots extract with different organic solvents as substitute of anthraquinone catalyst. Where: E.A., Ethyl acetate; CH_3_Cl, chloroform; DCM, Dichloromethane; P.E., Petroleum ether 60–80

This followed by petroleum ether extracts with screened yield (49.8%), and kappa number (22.1). Chloroform extractives obtained screened yield (45.4%) and admitted kappa number (24.9). Figure 2 and (Additional file 1: Table S1) showed that Ethyl acetate and ethanol crude extracts when used as substitute of AQ catalyst attained lowest screened yield (44.8% and 43.5%), respectively, with suitable kappa number (25.1 and 27.5), respectively. Also it could be noticed that the rejects ranged between (1.6% and 2.7%) with the exception of chloroform extract (4.6%) and as observed previously the screened yields increased and kappa numbers decreased according to the polarity of the solvents used. It could be noticed that *Sesamum Indicum* roots extracts have produced acceptable to satisfactory yields % and this due to the structure of the five anthrasesmonones that was found in the roots of *Sesamum Indicum* as mentioned by [3, 4] All of their structures have alkene chain in position 2 or 3. Adding the crude extracts of roots of *Sesamum Indicum* as AQ catalyst, this alkene chain may caused steric hindrance and prevents the reaction(to some extend) between the rest of AQ and the aryl ether bond or alkyl ether bond of side chain of lignin. This explicated the limited range of increasing screened yield by use the five organic solvent to extract the AQ. This improves the selectivity with respect to lignin removal without significant carbohydrates degradation.

Sugar industry is a strategic industry which high impact factor in national income White Nile factory is one of sugar factories in Sudan. Bagasse is fibrous matter that remains after sugar cane, for each ten tons of sugar cane crushed produced nearly three tones of wet bagasse, part of it used as biofuel. Unused bagasse was available on site in all of sugar factories causes’ environmental pollution and health complications (Additional file 1: Figure S6). Rational use of bagasse as source of pulp production instead of being an environmental pollutant.

## Limitations

The crude extracts of dichloromethane from *Sesamum Indicum* roots applied as Anthraquinone in pulping of bagasse indicated the suitability of this extract in chemical pulping. Although the results of pulping presented higher kappa number and reduced screened yield when compared with synthesized anthraquinone.

The purification of the crude extracts of the five solvents under study could provide suitable anthrasesamones as substitute of anthraquinones. The sodium hydroxide cooking of non woody plants, and agricultural residues with anthrasesamones improved the pulping yields and kappa number.

The study of the bagasse as by product in sugar industry, its utilization and its effect on environment and health should be done.

Application of purified extract with well-known chemical structure is highly needed. The analysis of extracts by spectral such as High Performance Liquid Chromatography, Ultraviolet–visible spectroscopy or ultraviolet–visible spectrophotometry, Fourier-transform infrared spectroscopy Nuclear magnetic resonance and electrospray ionization mass spectrometry is highly needed.

## Additional file

**Additional file 1: Figure S1.** White Nile Sugar Factory, White Nile State, Centre of Sudan (Source of Bagasse raw materials). **Figure S2.** Ethanol, ethyl acetate, chloroform, dichloromethane and petroleum ether of Roots of *S. indicum.* **Figure S3.** Pulp with dichloromethane extracts and sodium hydroxide of bagasse. **Figure S4.** Production of Sugar in White Nile Factory, White Nile state, Centre of Sudan. **Figure S5.** Amount of Bagasse as (byproduct unused bagasse) in White Nile Factory, White Nile State, and Centre of Sudan. **Figure S6.** Remains of Bagasse burned by sun rays and ash, causes environmental pollution and health complications. **Table S1.** Pulping properties for *S. indicum* roots extract with different organic solvents as anthraquinone catalyst.

## Abbreviations

AQ: anthraquinone
RDA: recommended daily allowance
AHQ: anthrahydroquinone
KOH: potassium hydroxide
Na_2_O: sodium oxide
TAPPI: Technical Association of Pulp and Paper Industry
