# Supplementary material for: Crude extracts of Sesamum Indicum roots used as anthraquinone source effect on pulping with sodium hydroxide of Sudanese bagasse
Source: BMC Res Notes. 2019 Jan 15;12:26. doi: 10.1186/s13104-019-4075-9 (PMC6334385; doi:10.1186/s13104-019-4075-9)
Supplement: Supplementary file 1 — Additional file 1: Figure S1. White Nile Sugar Factory, White Nile State, Centre of Sudan (Source of Bagasse raw materials). Figure S2. Ethanol, ethyl acetate, chloroform, dichloromethane and petroleum ether of Roots of S. indicum. Figure S3. Pulp with dichloromethane extracts and sodium hydroxide of bagasse. Figure S4. Production of Sugar in White Nile Factory, White Nile state, Centre of Sudan. Figure S5. Amount of Bagasse as (byproduct unused bagasse) in White Nile Factory, White Nile State, and Centre of Sudan. Figure S6. Remains of Bagasse burned by sun rays and ash, causes environmental pollution and health complications. Table S1. Pulping properties for S. indicum roots extract with different organic solvents as anthraquinone catalyst. [file 13104_2019_4075_MOESM1_ESM.docx]

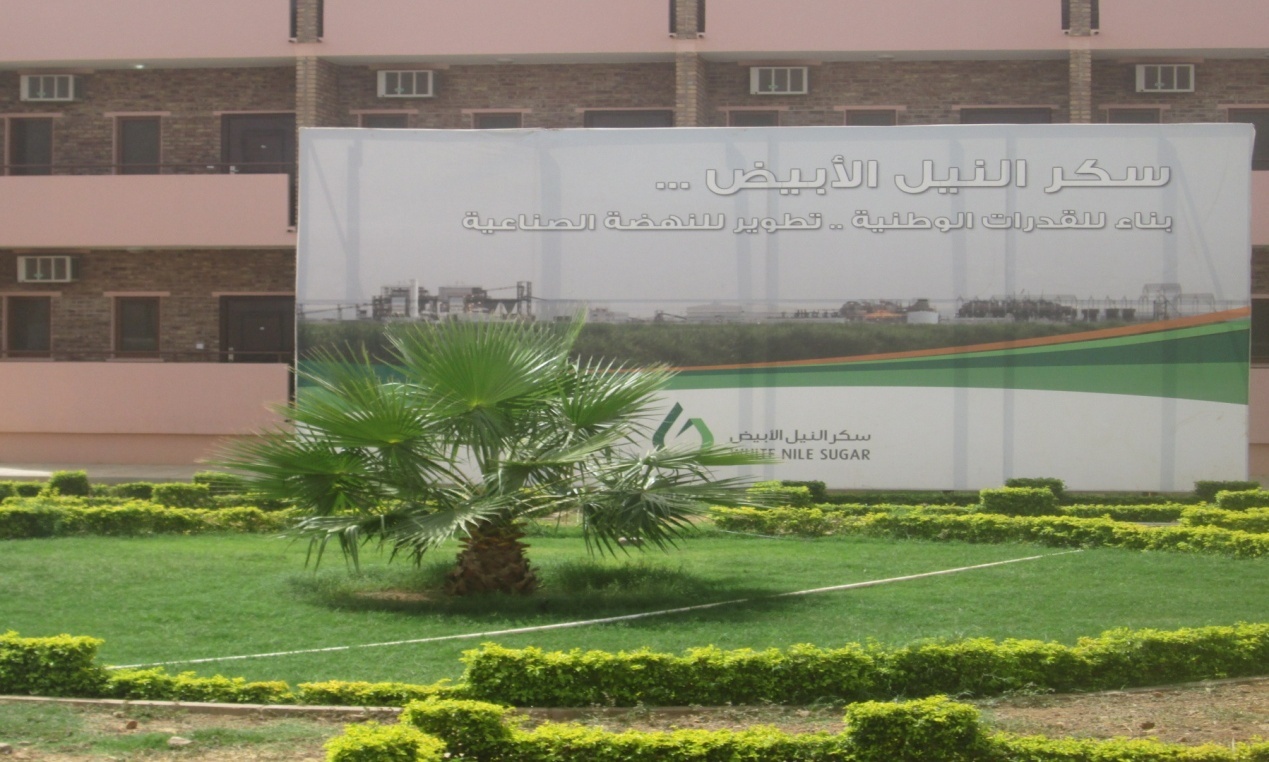


Additional file 1: Figure S1. White Nile Sugar Factory, White Nile State, Centre of Sudan (Source of Bagasse raw materials)


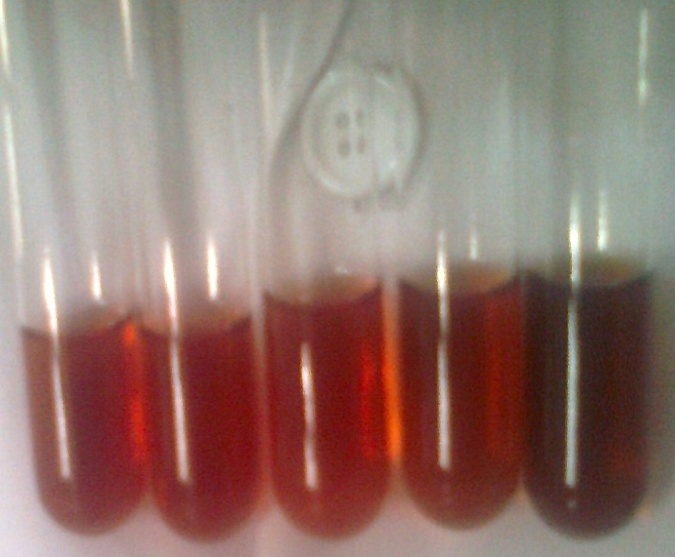


Additional file 1: Figure S2. Ethanol, ethyl acetate, chloroform, dichloromethane and petroleum ether of Roots of *S. indicum*


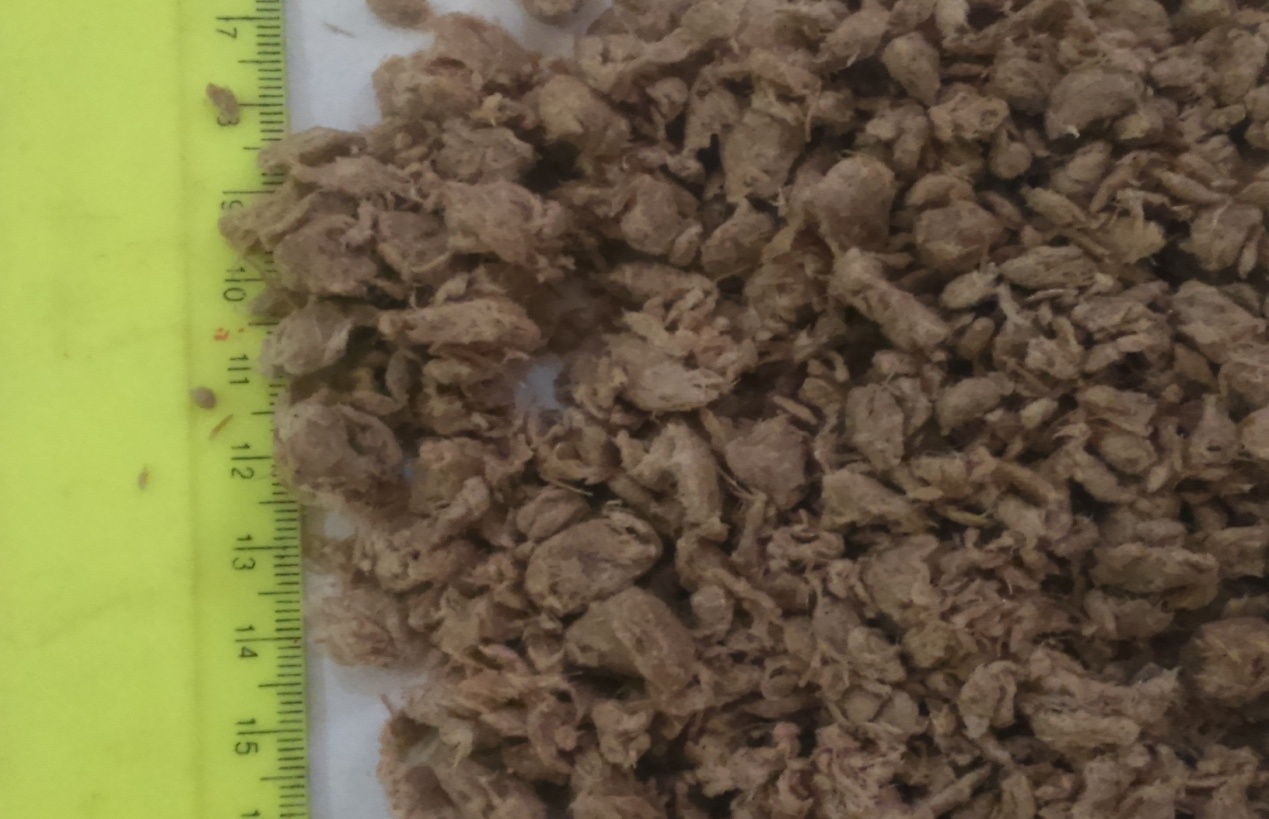


Additional file 1: Figure S3. Pulp with dichloromethane extracts and sodium hydroxide of bagasse.


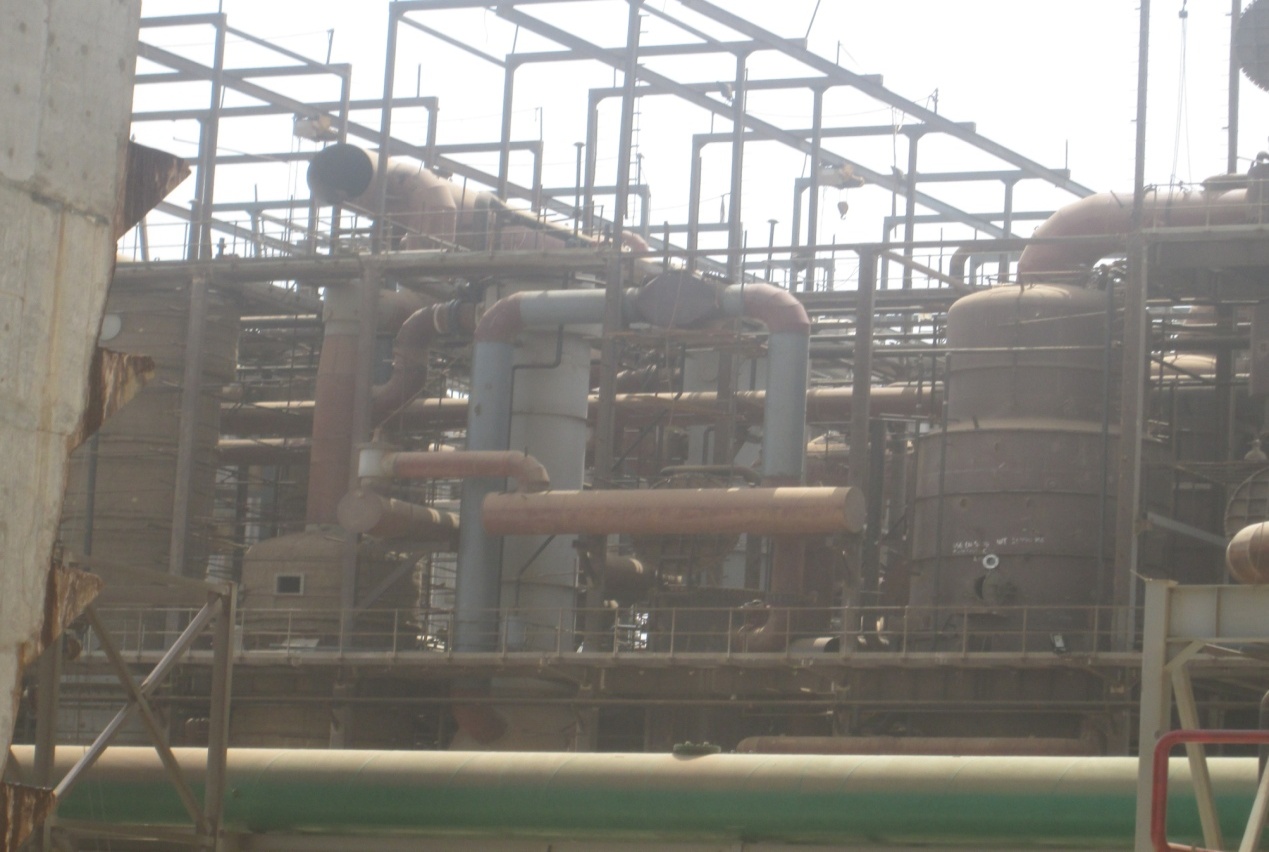


Additional file 1: Figure S4. Production of Sugar in White Nile Factory, White Nile state, Centre of Sudan


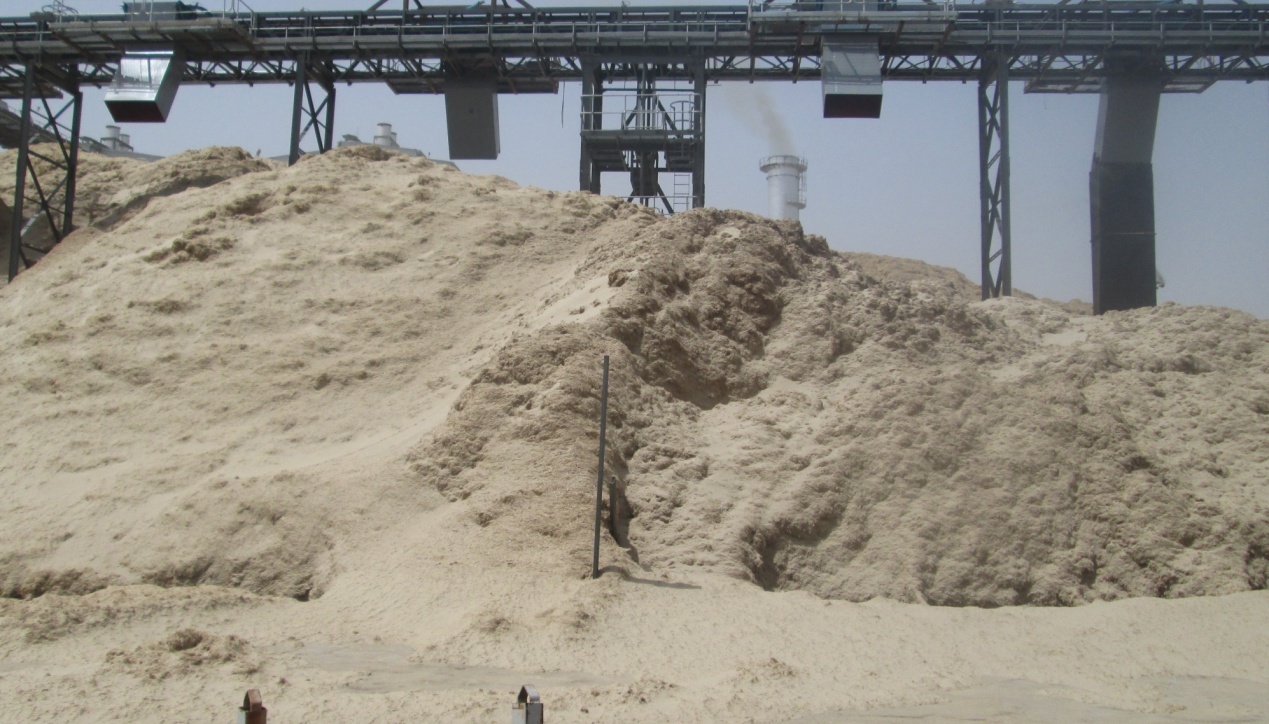


Additional file 1: Figure S5. Amount of Bagasse as (byproduct unused bagasse) in White Nile Factory, White Nile State, and Centre of Sudan


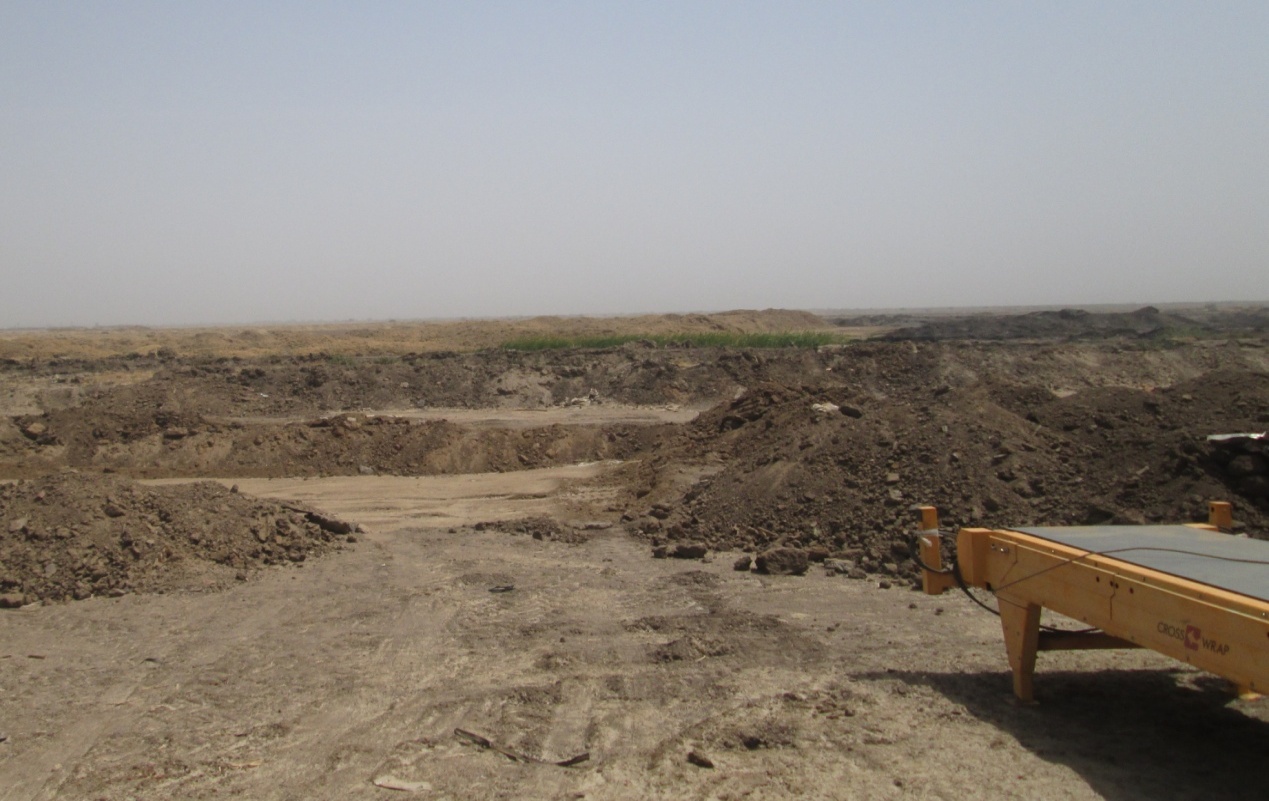


Additional file 1: Figure S6. Remains of Bagasse burned by sun rays and ash, causes environmental pollution and health complications

Additional file 1: Table S1. Pulping properties for *S. indicum* roots extract with different organic solvents as anthraquinone catalyst

| Roots Extractives | | | | |  |
| --- | --- | --- | --- | --- | --- |
| P.E | DCM | CH_3_Cl | E.A | Ethanol |  |
| 49.77 | 50.54 | 45.42 | 44.85 | 43.47 | Screened Yield % |
| 2.38 | 1.64 | 4.64 | 2.74 | 2.20 | Reject % |
| 52.15 | 52.18 | 50.06 | 47.59 | 45.68 | Total Yield % |
| 22.12 | 19.37 | 24.9 | 25.05 | 27.52 | Kappa No. |
